# Gender Incongruence and Autistic Traits: Cerebral and Behavioral Underpinnings

**DOI:** 10.1007/s10508-024-02809-5

**Published:** 2024-02-22

**Authors:** Behzad S. Khorashad, Yanlu Wang, Mats Holmberg, Cecilia Dhejne, Ivanka Savic

**Affiliations:** 1 Department of Women’s and Children’s Health, Karolinska Institute and University Hospital, 171 77 Stockholm, Sweden; 2 Department of Pediatrics, Susan B. Meister Child Health Evaluation and Research Center, University of Michigan Medical School, Ann Arbor, MI USA; 3 Radiology Unit, Department of Clinical Sciences, Intervention and Technology, Karolinska Institute, Stockholm, Sweden; 4 MR Physics Unit, Department of Medical Radiation Physics and Nuclear Medicine, Karolinska University Hospital, Stockholm, Sweden; 5 ANOVA, Karolinska University Hospital, Stockholm, Sweden; 6 Department of Medicine, Huddinge, Karolinska Institutet, Stockholm, Sweden; 7 Department of Neurology, University of California Los Angeles, Los Angeles, CA USA

**Keywords:** Gender incongruence, Cortical thickness, Social Responsiveness Scale, Autism, Body incongruence

## Abstract

Gender dysphoria and autism spectrum disorder (ASD) co-occur at high rates. Yet, it is unknown whether gender dysphoria and ASD are associated with common or distinct neurobiological correlates or how they relate to experiences of gender-related body incongruence. Using the Social Responsiveness Scale, we assessed autistic traits in 99 transgender and 99 cisgender individuals and investigated their associations with gender-related body incongruence, measured via a visually based “Body Morph” test, and with cortical thickness in the brain. Autistic traits were significantly higher among transgender individuals, and those with higher autistic traits had higher body incongruence scoring. Among transgender individuals, higher autistic traits were linked with a thinner cortex bilaterally in the temporal pole and the superior and inferior temporal gyri. Autistic traits were only partly associated with cortical morphology patterns previously reported in transgender individuals; instead, they were primarily linked to temporal lobe areas mediating social cognition. While replicating the previous literature on the increased prevalence of autistic traits among transgender individuals, this study reports specific regions in the brains of transgender individuals where cortical thickness is associated with autistic traits.

**Supplementary Information:**

The online version contains supplementary material available at 10.1007/s10508-024-02809-5.

## Introduction

There has been a growing interest in the co-occurrence of gender dysphoria and autism spectrum disorder (ASD) (Warrier et al., 2020). Gender dysphoria is defined as distress from incongruence between one’s experienced gender and one’s at-birth-assigned sex and a persistent strong desire to be of another gender (*Diagnostic and Statistical Manual of Mental Disorders*—DSM-5) (American Psychiatric Association, 2013). ASD is characterized by significant impairments in social communication and interactions—including difficulties in social-emotional reciprocity, nonverbal expression and understanding, understanding and maintaining social relationships—and restricted, repetitive patterns of behaviors, specific interests, and idiosyncratic sensory experiences (American Psychiatric Association, 2013; van der Miesen et al., 2018a, 2018b) (see the Supplementary Materials, Box 1, doi: 10.7302/21938).

Several studies have shown high co-occurrence rates between gender dysphoria and ASD (de Vries et al., 2010; van der Miesen et al., 2018a, 2018b; Warrier et al., 2020). Warrier et al. (2020) investigating cross-sectional datasets consisting of 641,860 individuals found that compared to cisgender individuals, transgender and gender-diverse individuals have, on average, higher rates of autism. de Vries et al. (2010) found that the incidence of ASD among those with gender dysphoria is as eight times higher than in the general population, and van der Miesen et al. (2018a, 2018b) found that the wish to be of the opposite gender is significantly higher among adolescents (6.5%) and adults (11.4%) with ASD as compared to the general population (3–5%). Most recently, a systematic review on ASD and gender dysphoria/incongruence demonstrated a pooled prevalence estimates of ASD diagnoses in those with gender dysphoria of 11% and higher degrees of autistic traits in those with compared to without gender dysphoria but concluded that the extent of this link needs further investigation (Kallitsounaki & Williams, 2023).

Although emerging evidence supports an association between ASD and gender dysphoria, the underlying determinants for this co-occurrence are unclear. One possibility is that gender incongruence and gender dysphoria could be a result of primary autistic traits; social communication challenges might result in experiences of not fitting in with members of their own gender, and conclusions that they might better fit in with those of the opposite gender (Pasterski et al., 2014). Alternatively, autistic traits could be secondary to gender dysphoria; discrimination and stigma could cause social impairment and deficits in individuals with gender dysphoria, which could appear as autistic traits (Turban & van Schalkwyk, 2018). An understanding of the mechanism underlying the co-occurrence of ASD/ASD traits and gender dysphoria has clinical relevance, particularly in the perspective of gender-affirming treatments (Almazan & Keuroghlian, 2021; Baker et al., 2021; Matthys et al., 2021).

Both for gender dysphoria and for ASD, cortical thickness has been a focus of interest (Burke et al., 2017; Mueller et al., 2021) or ASD (Yang et al., 2016; Zielinski et al., 2014). Few studies have, however, investigated associations between cortical thickness and dimensional measures of gender dysphoria, such as the Body Morph Index (Feusner et al., 2016; Wang et al., 2021), and between cortical thickness and dimensional measures of autistic traits such as Social Responsiveness Scale (SRS) scores (Tu et al., 2016). Such studies are of interest as they could provide additional information about a possible link between cortical thickness, gender dysphoria, and ASD. In the present study we, therefore, specifically investigated how, and whether autistic traits in individuals with gender dysphoria are related to cortical thickness.

The aims were threefold. Firstly, we tested whether we could replicate the previous findings of increased autistic traits in transgender compared to cisgender individuals. Secondly, we aim to investigate the associations between the autistic traits and gender dysphoria in more detail, via measuring the degree of correlation between two known quantitative measures of gender dysphoria and autistic traits; and lastly, we aim to explore the relationship of cortical thickness with autistic traits. In particular, we investigate whether adding a quantified measure of autistics traits can affect the regions previously shown to differ between transgender and cisgender individuals. Based on previous studies (Heylens et al., 2018; Skagerberg et al., 2015; van der Miesen et al., 2018a, 2018b), we hypothesized that autistic traits, indexed by the SRS score, would be higher in transgender participants than in cisgender participants; and that SRS scores would be associated with the scoring in the Body Morph Index (i.e., higher SRS scores would be associated with lower Body Morph Index scores, reflecting gender dysphoria). Aim 3 was exploratory.

## Method

### Participants

We carried out a combined behavioral and imaging analysis examining autistic traits in a total of 199 binary transgender and cisgender individuals who participated in a two-site study in Stockholm, Sweden and Los Angeles, USA. All participants had not yet received gender-affirming treatments, and none were given any neuropsychiatric diagnosis. Table 1 shows their demographics, autistic traits (SRS scores), and Body Morph Index scores. Recruitment at the Stockholm site has been explained in detail elsewhere (Manzouri & Savic, 2019). Recruitment from the UCLA site occurred in the community through flyers and website advertisements and included outreach to transgender resource centers in Los Angeles County, Ventura County, and Orange County, as well as clinics at UCLA and in the community that provide transgender healthcare.

**Table 1:** Demographic characteristics, Body Morph Index, and Social Responsiveness Scale scores of participants

	Cisgender			Transgender			Total		Sex^a^	Gender^a^
	Female	Male	Total	Female	Male	Total	Female	Male		
*Age (in years)*									*p* = .034	*p* < .001
*N*	53	46	99	61	39	100	114	85		
Mean (SD)	29.64 (7.4)	29.52 (6.63)	**29.59 (7.02)**	23.97 (5.91)	26.21 (4.82)	**24.84 (5.59)**	**26.61 (7.2)**	**28 (6.07)**		
Min	19	19	19	18	19	18	18	19		
Max	45	44	45	42	37	42	45	44		
*Years of education*									NS	*p* < .001
*N*	51	42	93	60	37	97	111	79		
Mean (SD)	16.47 (3.09)	16.05 (2.66)	**16.28 (2.9)**	13.74 (1.93)	14.42 (2.24)	**14 (2.07)**	15 (2.87)	15.28 (2.59)		
Min	12	11	11	9	10	9	9	10		
Max	26	24	26	17.5	20	20	26	24		
*Body Morph Index (short exposure)*									NS	*p* < .001
*N*	22	15	37	40	19	59	62	34		
Mean (SD)	30.8 (12.36)	13.46 (29.61)	**23.77 (22.46**)	− 8.71 (28.4)	− 6.74 (28.65)	− **8.07 (28.25)**	5.31 (30.52)	2.17 (30.39)		
Min	7	− 41	− 41	− 80	− 46	− 80	− 80	− 46		
Max	58.66	53.5	58.66	61.425	57.46667	61.425	61.425	57.46667		
Body Morph Index (long exposure)									NS	*p* < .001
*N*	22	15	37	40	18	58	62	33		
Mean (SD)	33.88 (13.12)	19.82 (36.27)	**28.18 (25.71)**	− 8.04 (57.3)	− 10.69 (48.96)	− **8.86 (54.43)**	6.83 (50.67)	3.18 (45.68)		
Min	9	− 37	− 37	− 240	− 95	− 240	− 240	− 95		
Max	57.84	65.06	65.06	152.5	100	152.5	152.5	100		
Social Responsiveness Scale (total score)									*p* = .031	*p* < .001
*N*	53	46	99	61	38	99	114	84		
Mean (SD)	46.70 (8.06)	44.17 (8.11)	**45.53 (8.14)**	53.23 (12.69)	51.42 (12.83)	**52.54 (12.71)**	**50.19 (11.23)**	**47.45 (11.06)**		
Min	67	74	74	111	90	111	111	90		
Max	35	34	34	37	36	36	35	34		

^a^Statistical significance for the main effect of at-birth-assigned sex and gender

Inclusion criteria for the transgender participants were adults aged 18–50 who were given the diagnosis of “Transsexualism” based on diagnostic criteria of ICD-10 (F64.0; World Health Organization, 2019), or gender dysphoria (DSM-5) (American Psychiatric Association, 2013). Because accepted nomenclature has changed since the study began (Bouman et al., 2017), and to be in line with DSM-5 terminology, we will refer to our participants as “transgender participants” and the comparison group, as “cisgender participants” (See Box 1 for more details). Exclusion criteria for the transgender participants included previous or current hormonal treatment or gender-affirming surgery at the time of the first scanning session, or any known chromosomal or hormonal disorder. Exclusion criteria for both groups included a previous diagnosis of severe ASD (see Box 1), any current psychiatric or substance use disorder, any neurological or other major medical disorder, or the use of any medications with psychotropic effects.

### Measures

#### Social Responsiveness Scale (SRS)

The Social Responsiveness Scale is a 65-item questionnaire completed by parents or someone close to the participants and designed to provide a quantitative assessment of autistic traits in addition to characterization of individual autistic impairments (Constantino, 2013). The SRS has shown a sensitivity of .80 and a specificity of .69 for autism and correlated significantly with the social domain (*r* = 0.35) and communication domain (*r* = .32) subscores of the Autism Diagnostic Observation Schedule (ADOS), which is widely considered as the gold standard for autism diagnosis (Bölte et al., 2011). It should be emphasized that the strength of the correlation of SRS with the ADOS is not necessarily highly relevant to our study since it is measuring traits in those without a prior diagnosis of autism. Each item of the SRS contains a statement, for which its applicability to the participant is assessed using a scale ranging from 0 to 3. The SRS includes five subscales: social awareness (e.g., “Knows when he/she is too close to someone or invading someone’s space”), social cognition (e.g., “Concentrates too much on parts of things rather than ‘seeing the whole picture’[for example, if asked to describe what happened in a story, child may talk only about the kind of clothes the characters were wearing]”), social communication (e.g., “When under stress, child seems to go on ‘auto-pilot’ [for example, shows rigid or inflexible patterns of behavior]”, social motivation (e.g., “Does not join group activities unless told to do so”), and autistic mannerisms (e.g., “Has repetitive, odd behaviors, such as hand flapping or rocking”). Raw total scores, obtained from the instrument, are then converted to at-birth-assigned sex-normed *T* scores following instructions provided by authors (Constantino, 2013).

Since the adult version of SRS had not been validated in the Swedish population when the study commenced, we used the original SRS (Swedish translation) that was designed for children. To maintain consistency, the identical questionnaire was administered to the sample in the US participants’ parents or individuals closely acquainted with the participant, who had known them from childhood to adulthood, and were requested to respond to inquiries concerning the participant's adulthood. Therefore, the following four items on the SRS that reference childhood were excluded when calculating the final score: 9. Clings to adults, seems too dependent on them; 22. Plays appropriately with children his/her age; 24. Has more difficulty than other children with changes in his/her routine; and 43. Separates easily from caregivers. Items 9 and 43 are used in calculation of social motivation subscale, item 22 in social communication subscale, and item 24 in autistic mannerism. The Cronbach alpha of the Social Responsiveness Scale was .908.

#### Body Morph Task

The “Body Morph” task, as detailed in Burke et al. (2019), was designed to explore perceptions of the body and experiences related to gender identity. It aims to capture gender-related body incongruence. In addition to the verbally expressed incongruence, as measured via instruments such as Transgender Congruence Scale, with which the Body Morph Index has been shown to be positively associated (Moody et al., 2020), body morph task as a visually based tool, can further help achieve a quantitative estimate of dysphoria/ incongruence. For the body morph task, each participant is required to dress in a form-fitting full body unitard to provide an accurate representation of their body shape without the discomfort of being nude. Hands, feet, and head were cropped from the photos, and both front and side views were taken. Each participant’s picture was morphed toward those pictures of five different female-presenting and five different male-presenting individuals at degree intervals of 20%, using FantaMorph Software, version 5.0 (http://www.fantamorph.com). Eleven morph conditions resulted, ranging between − 100% morphed completely to a picture of a cisgender person with opposite birth-assigned sex and + 100% morphed completely toward a picture of a cisgender person with same birth-assigned sex as the participant. Thus, 0% referred to the original unmorphed own-body image of the participant. A set of 62 images (using a randomized order and number of repetitions of the body image morphs and unmorphed own-body image) were presented for two different viewing conditions (short duration = .5 s and long duration = 2 s), totaling 128 trials. These images were presented using MATLAB 2012a, on a laptop computer. Each trial consisted of the image (presented for either .5 or 2 s) followed by a 1 s response screen with button press options, followed by a fixation cross. Participants were instructed to respond as quickly as possible to the question “To what degree is this picture you?” on a 4-point scale (1: 0–25% “me”; 2: 25–50% “me”; 3: 50–75% “me”; and 4: 75–100% “me”). We calculated the “body index” (BI): the perception of the degree of self, represented by an index calculated from ratings across all of the morphed bodies presented. The body index provides an indication of an individual’s maximal perception of ‘self’ on a continuum from traditionally feminine to traditionally masculine appearances. The Body Morph Index score was calculated from the body morph task ratings by multiplying each degree of “self” rated for each morph with the morph degree (20%, 40%, 60%, 80%, or 100%) for each image (Feusner et al., 2017) (Figure S1, doi: 10.7302/21938). Weighted values were averaged for each participant across all image ratings and divided by the number of rated images. This provided an average index, the Body Morph Index, of self-perception for each participant, weighted by how close or far from the actual self-photograph the image was morphed, and in which direction. Thus, across image ratings, positive mean Body Morph Index values represent higher identification with bodies morphed toward one’s birth-assigned sex (suggesting gender congruency), while negative mean body morph values represent higher identification with bodies morphed opposite to one’s toward birth-assigned sex (suggesting gender incongruency).

#### MRI Data Acquisition

In Stockholm, magnetic resonance imaging (MRI) data were acquired on a 3-Tesla MRI scanner (Discovery 3 T GE-MR750, General Electric, Milwaukee, WI) equipped with an 8-channel phased array receiving coil. T1-weighted images were acquired with a Spoiled Gradient Echo Pulse (SPGR) sequence with following parameters: 1 mm isotropic voxel size; TR = 7.9 ms; TE = 3.1 ms; FoV = 23 cm; flip angle = 12°. In Los Angeles, MRI data were acquired on a 3-Tesla Siemens Prisma scanner with a 32-channel head coil. T1-weighted images were acquired with a Magnetization Prepared Rapid Acquisition Gradient Echo (MPRAGE) sequence with the following parameters: 1 mm isotropic voxel size; distribution factor 50%; TR = 1900 ms; TE = 3.26 ms; flip angle = 9°. Before initiation of this collaborative study, we carefully harmonized data acquisition parameters to minimize effects of scanner difference.

#### Cortical Thickness Analyses

Cortical reconstruction was performed using the FreeSurfer image analysis suite, version 6.0.0 (Fischl & Dale, 2000) to derive measures of cortical thickness and total intracranial volume (TIV). T1-weighted images were processed using FreeSurfer software (version 6.0.0), which included skull stripping, Talairach transforms, atlas registration, spherical surface maps, and parcellations. The resulting images were visually inspected for accuracy for all participants and were manually edited when needed (primarily to improve skull stripping). Images from 30 individuals (14 controls) needed manual edition, but all gained sufficient quality to be included in the study and none were excluded.

### Statistical Analyses

All descriptive statistics, group comparisons and correlation analysis were done in SPSS Statistics 25 (SPSS Inc., Chicago, IL). Initial exploration of the data indicated that the SRS scores were not normally distributed, as confirmed by Shapiro–Wilk tests and visual inspection of histograms. To achieve normality, a Box-Cox transformation was applied within SPSS. Statistical analyses were conducted to answer four separate questions.

Question 1: Is there any difference between transgender and cisgender participants in their SRS scores and its subscales? Univariate regression analysis was conducted to examine the main effect of gender (cis vs. trans) and at-birth-assigned sex (male versus female) on the SRS total scores as well as the subscales. We also examined the data for outliers which were defined as observations that deviate than two standard deviations from the mean values.

Question 2: Are SRS scores and Body Morph Index (short exposure) scores associated? We analyzed the correlation between these two sets of scores among all participants, and then separately in transgender and cisgender participants. In each sample, Pearson correlation coefficients were calculated to assess the relationships between the two variables. To obtain more robust estimates and confidence intervals, bootstrapping techniques were employed. Specifically, 1,000 bootstrap resamples were generated to calculate 95% confidence intervals for each correlation coefficient.

Question 3: Are SRS scores (total score and its subscales) associated with cortical thickness, in any specific region of the brain? In order to answer this question, using FreeSurfer software, a general linear model (GLM) was used to analyze the association between cortical thickness and total SRS scores using age and site of recruitment/scanner as covariates of no interest, among transgender and cisgender participants separately. Cortical thickness data were smoothed by applying a two-dimensional Gaussian smoothing kernel of 10 mm. To correct for multiple comparisons, a Monte Carlo correction was performed. Only clusters with a significance threshold of *p* < .05 and minimum cluster size of 10 cm^2^ at the cluster level were reported. For illustration purposes, the mean cortical thickness for each of the significant cluster(s) were also extracted (if there was any) and the correlation between mean cortical thickness in that brain region and Social Responsiveness Scale scores were reported.

Question 4: Are regional cortical thickness differences between transgender and cisgender individuals explained by the degree of autistic traits as measured by Social Responsiveness Scale scores (total score)? In order to answer this question, we first compared the cortical thickness between transgender and cisgender participants using a GLM in FreeSurfer with age and site of recruitment as covariates of no interest. We then repeated the analysis but this time with Social Responsiveness Scale total scores added as another covariate of no interest. Cortical thickness data were smoothed by applying a two-dimensional Gaussian smoothing kernel of 10 mm. To correct for multiple comparisons, a Monte Carlo correction was performed. Only clusters with a significance threshold of *p* < .05 and minimum cluster size of 10 cm^2^ at the cluster level were reported.

## Results

### Demographics

The mean (± standard deviation) age in the transgender sample (*n* = 100) was 24.84 (± 5.59), which was significantly lower compared to the mean age 29.59 (± 7.02) in the cisgender sample (*n* = 99). This was also the case for the number of years of education, which in the transgender sample was 14.00 (± 2.07) and in the cisgender sample was 16.28 (± 2.90). There were no differences between the participants recruited from KI and UCLA in mean age (KI, 27.6 ± 6.99; UCLA, 25.71 ± 5.64) or years of education (KI, 15.24 ± 2.87; UCLA: 14.55 ± 2.05) (Table 1). One transgender man participant lacked the completed SRS scores, so a total number of 198 participants with completed SRS (99 transgender and 99 cisgender participants) were analyzed. Out of these 198 participants, Body Morph Index (short and long exposure) was available for 96 (59 transgender and 37 cisgender) participants. One transwoman lacked the score for Body Morph Index (long exposure).

Question 1: Is there any difference between transgender and cisgender participants in their SRS scores and its subscales?

**Fig. 1:**
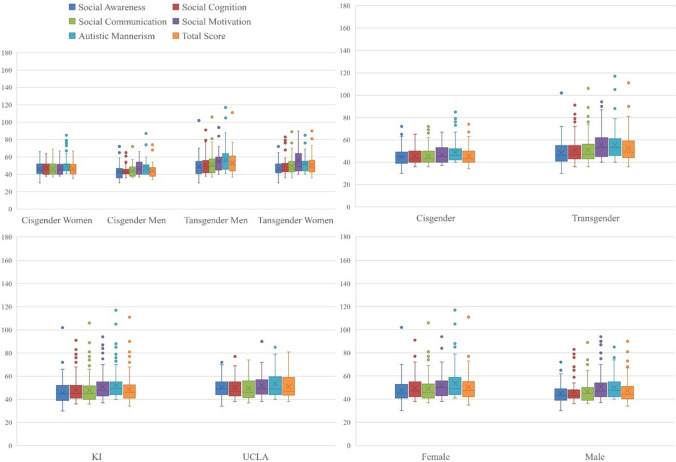
Distribution of Social Responsiveness Scale (total score) and its subscales among the participants based on the study group, site of recruitment, at-birth-assigned sex, and gender identity. In all subscales, transgender participants had significantly higher scores compared to cisgender participants (SocAw, *p* = .021; for all the rest *p* < .001). At-birth-assigned females had significantly higher scores in Social Cognition (*p* = .007), Autistic Mannerism (*p* = .018) and total SRS score (*p* = .031). Participants recruited from UCLA had significantly higher scores than those recruited from KI only on the Social Awareness subscale (*p* = .002). KI, Karolinska Institute; UCLA, University of California Los Angeles

SRS total score was not normally distributed, with a skewness of 1.8 (± .17) and kurtosis of 5.08 (± .34). After applying the Box-Cox transformation, the skewness was reduced to .11 (± .17), and the kurtosis to − .25 (± .34). There was a significant main effect of gender identity (*F* = 23.13, *p* < .001) on the SRS total score, where transgender participants (*n* = 99, 52.54 ± 12.71) had generally higher scores than cisgender participants (*n* = 99, 45.53 ± 8.14). The difference for at-birth-assigned sex was not significantly different [*F* = 3.13, *p* = .078, at-birth-assigned females (n = 99) scored 50.19 (± 11.23) and at birth-assigned males (*n* = 85) scored 47.45 (± 11.06)] (Table 1, Fig. 1). All the subscales of SRS were significantly higher among transgender participants compared to cisgender participants. There was marginally significant difference between participants recruited from KI and UCLA [*t*(195) = − 1.99, *p* = .047). Table S1 provides more details on participants’ SRS total and subscale scores. Although participants had been screened for not having severe ASD, in the case of six transgender participants (three transmen and three transwomen), the SRS total scores were in the clinically significant range (> 76).

Question 2: Are Social Responsiveness Scale scores and Body Morph Index (short exposure) scores associated?

**Fig. 2:**
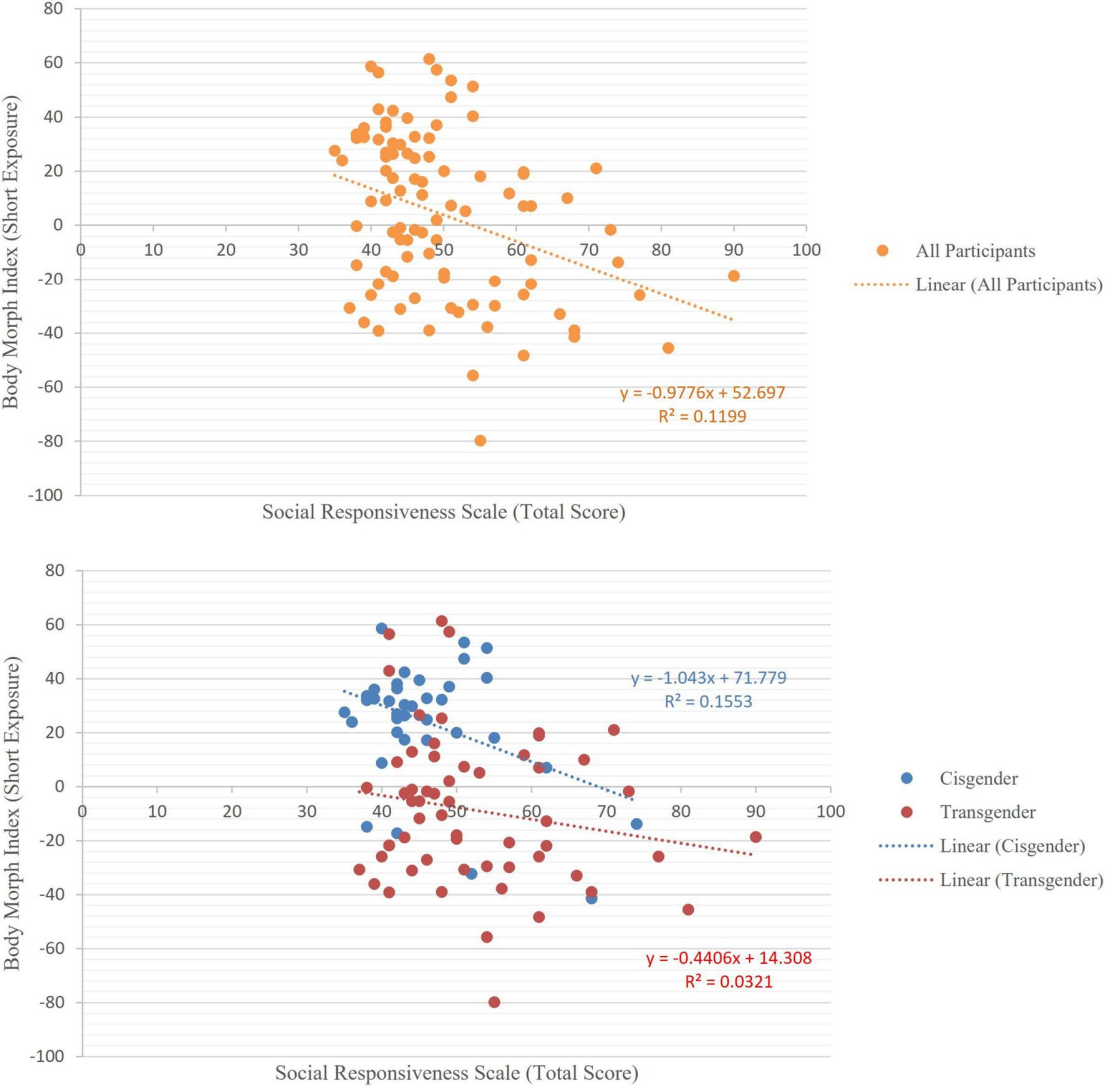
Correlation of Social Responsiveness Scale (total *T* scores) and Body Morph Index (short exposure) across all participants (*n* = 95, above), all transgender participants (*n* = 58) and all cisgender participants (*n* = 37) (below). A significant and negative correlation was found between the SRS total scores and Body Morph Index among all participants (*r* (95) = − .329, *P* = .001), indicating that those with higher autistic traits had greater body incongruence. The correlation was negative in both transgender and cisgender participants separately, but not statistically significant

The correlation of Body Morph Index (short and long exposure) with Social Responsiveness Scale total score was calculated among all participants using bootstrapped Pearson correlation. For Social Responsiveness Scale total score and Body Morph Index (short exposure), the Pearson correlation coefficient was found to be *r*(95) = − .325, *p* = .001. Bootstrapping with 1000 resamples was used to estimate the 95% confidence interval for the Pearson correlation coefficient (− .485 to − .153) (Fig. 2). For long exposure, the Pearson correlation coefficient was found to be *r*(94) = − .241, *p* = .019, 95% interval of − .4 to .05. The observation that Body Morph Index was significantly and negatively correlated with Social Responsiveness Scale total scores means that those with higher autistic traits were more identified with bodies morphed opposite to their at-birth-assigned sex (suggesting greater gender incongruency). The details of correlation coefficients for Social Responsiveness Scale subscales can be found in Table S3.

Data from nine participants (four transgender men, four transgender women and one cisgender man) were found to be potentially outliers as they had Social Responsiveness Scale total scores higher than 2 standard deviations from the mean. However, after Box-Cox transformation, no outlier was found. We also analyzed the correlation between Social Responsiveness Scale total scores and Body Morph Index (short and long) among subsamples of all transgender participants [short: *r*(57) = − .165, ns; long: *r*(57) = − .085, ns] and all cisgender participants [short: *r*(37) = − 0.276, ns; long: *r*(37) = − 0.349, *p* = 0.034]. The distribution of Social Responsiveness Scale total scores and Body Morph Index in cisgender and transgender populations are shown separately in figure S3.

**Fig. 3:**
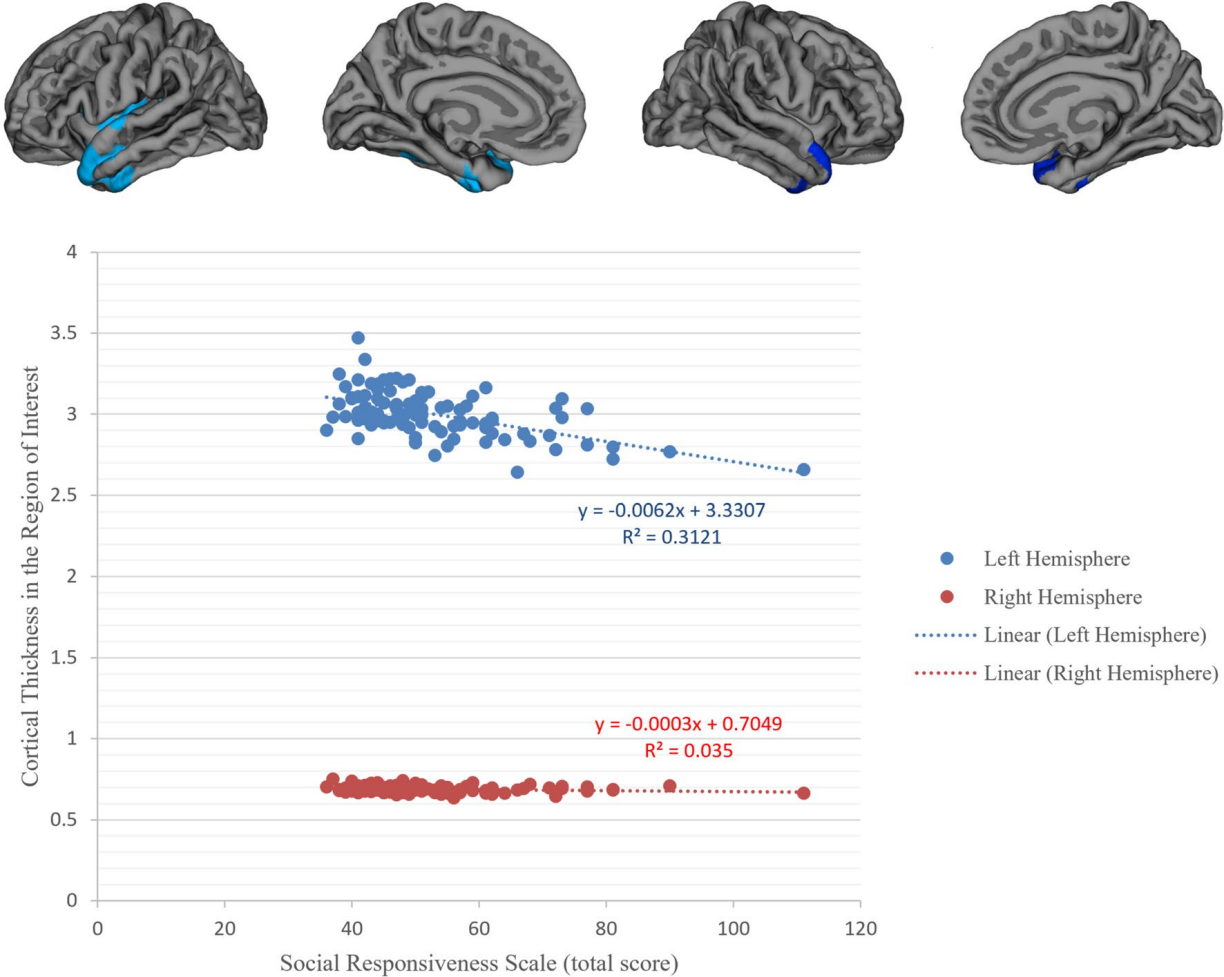
Clusters exhibiting statistically significant correlation with Social Responsiveness Scale (SRS) (total *T* scores) among transgender participants (above). Results were corrected for multiple comparisons using Cluster-based Monte Carlo simulations, *p* < .05. The mean cortical thickness at all cluster in left and right hemisphere was extracted, and their correlation against SRS total *T* scores is illustrated (below). The SRS total *T* scores were significantly and negatively correlated with cortical thickness in left superior temporal (size 47, *X*: − 42.7; *Y*: 10; *Z*: − 22.5) (*r* = − 0.5, *p* < 0.001) but only at trend level with the cluster in the right superior temporal (size 1082.33; *X*: 46.9; *Y*: 11.1; *Z*: − 18.9) (*r* = − 0.184, *p* = 0.069). When removing the outliers, the correlations became significant for both left (*r* = − 0.462, *P* < 0.001) and right (*r* = − 0.247, *P* = 0.018) hemispheres

Question 3: Are Social Responsiveness Scale scores (total score and its subscales) associated with cortical thickness, in any specific region of the brain, in transgender participants? Social Responsiveness Scale total scores (Fig. 3) and all its subdomain scores (Figure S2 and Table S2) were significantly and negatively correlated with cortical thickness in left and (in some subscales) right superior temporal, inferior temporal gyri, and the temporal pole. Thus, those with higher autistic traits had thinner cortex in these regions. Corresponding analyses in cisgender participants revealed no significant clusters.

**Fig. 4:**
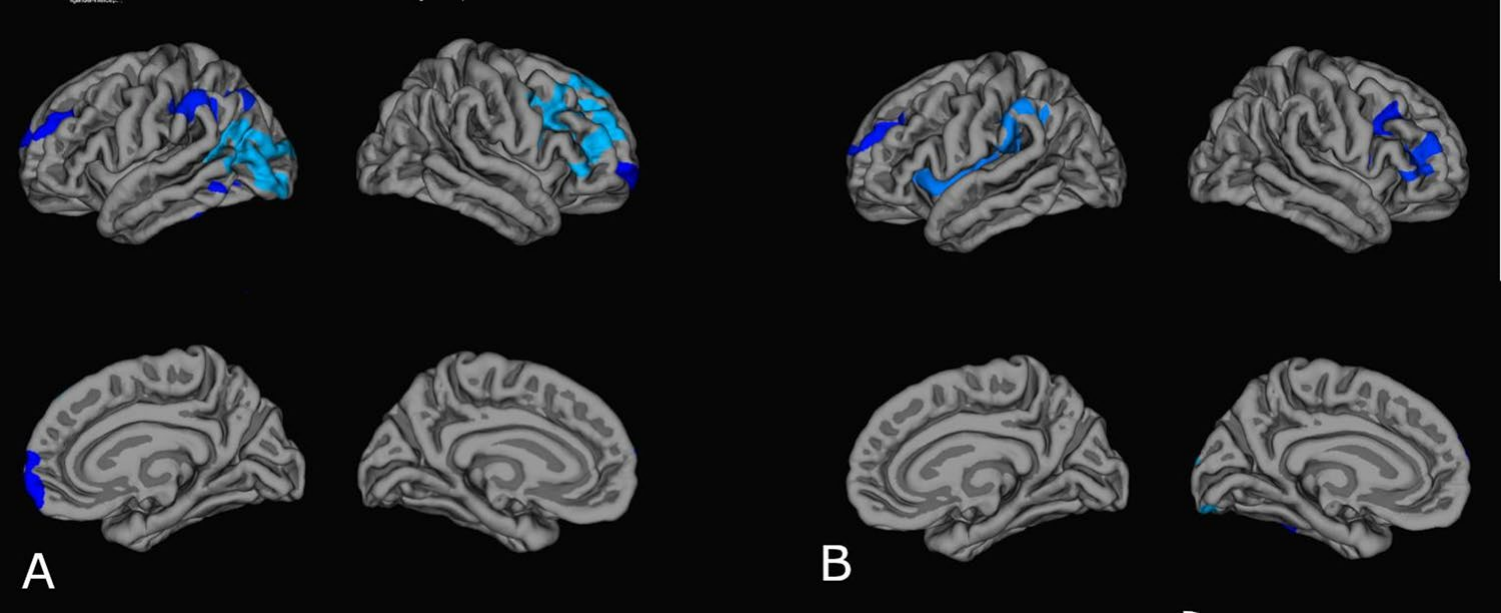
Clusters exhibiting significant differences in cortical thickness between cisgender participants and transgender participants. Results were corrected for multiple comparisons using cluster-based Monte Carlo simulations, *p* < 0.05. **A** Cisgender–transgender comparison with age and site of recruitment as covariates of nuisance: Left inferior parietal (*x* = − 32.4, *y* = − 51.9, *z* = 35), left supramarginal (*x* = − 55.3, *y* = − 31.6, *z* = 39.4), left middle temporal (*x* = − 46.3, *y* = − 59.1, *z* = 7.6), left inferior temporal (*x* = − 44.8, *y* = − 54.0, *z* =− 4.0), left rostral middle frontal (*x* = − 22.0, *y* = 44.4, *z* = 22.5), right caudal middle frontal (*x* = 37.8, *y* = 20.3, *z* = 30.3), right rostral middle frontal (*x* = 21.5, *y* = 54.4, *z* = − 3.9); **B** Cisgender–transgender comparison with age, site of recruitment and SRS total *T*-score as covariates of nuisance: left superior temporal (*x* = − 41.4, *y* = − 34.3, *z* = 8.0), left insula (*x* = − 36.3, *y* = 3.7, *z* = 1.0), left rostral middle frontal (*x* = − 21.8, *y* = 48.4, *z* = 20.7), right caudal middle frontal (*x* = 38.2, *y* = 21.1, *z* = 32.0), right caudal middle frontal (*x* = 38.2, *y* = 21.1, *z* = 32.0)

Question 4: Are regional cortical thickness differences between transgender and cisgender individuals explained by the degree of autistic traits as measured by Social Responsiveness Scale scores (total score)? The first comparison of cortical thickness between transgender and cisgender participants using age and site of recruitment as covariates of no interest showed thicker cortex in transgender participants in left inferior parietal, left supramarginal, left middle temporal, left inferior temporal, left rostral middle frontal, right caudal middle frontal, and right rostral middle frontal gyri (Fig. 4A). The second comparison, after adding SRS total scores as an additional covariate of no interest, yielded significant clusters in the left superior temporal cortex, left insular cortex, left rostral middle frontal cortex, right caudal middle frontal cortex, and the right caudal middle frontal cortex (Fig. 4B). The parietal lobe clusters (left hemisphere) and the frontal lobe clusters (right hemisphere) were, however, considerably smaller when regressing out the SRS scores; this could suggest a link to autistic traits, although simple loss of power with an additional covariate cannot be ruled out.

## Discussion

This study quantified relationships between autistic traits, body incongruence, and brain cortical morphological features in transgender and cisgender individuals. Autistic traits were higher in transgender participants compared with cisgender controls. Across all participants, those with higher autistic traits also had higher body incongruence. Including new participants from another recruitment site (Los Angeles), we replicated our previous finding of greater cortical thickness in transgender participants compared to cisgender participants in cerebral midline regions and in the left parietal cortex. Yet, autistic traits did not correlate with cortical thickness in regions found to differ between the groups. Instead, autistic traits were inversely correlated with cortical thickness in temporal lobe areas known to mediate social awareness and socio-cognitive functions (Saitovitch et al., 2012). The observation that the transgender individuals had higher scoring *across all* SRS subscales, including manneristic behaviors and not just limited to social characteristics, is in line with several previous reports (Akgül et al., 2018; Russell et al., 2021; Skagerberg et al., 2015) and suggests that higher autistic traits are not limited to social domains and thus not secondary to the social consequences (van der Miesen et al., 2018a, 2018b).

To the best of our knowledge, this is the first study to examine associations between autistic traits and cortical thickness in a sample with gender dysphoria. Associations between cortical thickness and autistic traits (measured by SRS scores) have, however, been reported in individuals with ASD as well as in neurotypical individuals (Braden & Riecken, 2019; Prigge et al., 2018; Tu et al., 2016; Yang et al., 2016). In adult patients with ASD, higher SRS scores were found to be associated with thinning of the temporal lobe cortex (Braden & Riecken, 2019; Prigge et al., 2018; Tu et al., 2016; Zielinski et al., 2014). For example, Tu et al. (2016) reported association between higher SRS total scores and thinner right superior temporal and also bilateral insular cortex (Tu et al., 2016), whereas Prigge et al. (2018) found that SRS raw total scores in male individuals with ASD were positively correlated with cortical thickness in the right frontal and temporal pole, and negatively correlated with cortical thickness in the right posterior cingulate and left inferior temporal gyrus (Prigge et al., 2018). Our findings replicated the negative correlation in the left inferior temporal gyrus but not the positive correlation in the temporal pole.

Several groups have found that this regional cortical thinning shows a steeper age-related slope from child and adulthood among individuals with ASD compared with neurotypical controls. A cross-sectional study suggested that in individuals with ASD, compared to controls, cortical thickness goes through faster age-related thinning in the left pars opercularis, the left temporal cortex, the left inferior parietal lobule, and the left lateral occipital lobe (Braden & Riecken, 2019). It should be noted that our transgender participants had not received ASD diagnoses, as this was an exclusion criterion. This might explain the observation that, rather than a significant thinning of the temporal lobe cortex, we detected a negative correlation between autism traits and cortical thickness in the superior temporal gyrus. Another important observation was that autism traits were *not* correlated to what we previously described as increased cortical thickness along the cerebral midline.

One way for addressing the etiology of autistic traits and gender dysphoria in future studies is through longitudinal analyses examining whether and how increased own-body self-congruence following gender-affirming hormone treatment is linked to changes in associations with autistic traits. The literature thus far suggests that there is no link; see Hogstrom et al. (2013), Klein et al. (2014), and Kohli et al. (2019). A further approach would be to extend the study population and categorize transgender participants into two subgroups: high and low autistic traits—assuming that the social stigma of being transgender with gender dysphoria is similarly distributed in both groups. At present, we can only conclude that SRS scores are elevated in persons with gender dysphoria, and that these autistic traits are associated with cortical thickness characteristics previously associated with ASD.

### Methodological Considerations and Future Studies

One limitation is that, given an ASD diagnosis was an exclusion criterion, the results cannot necessarily be generalized to those given both a gender dysphoria and an ASD diagnosis. Another limitation is the cross-sectional nature of this study; longitudinal studies that follow individuals from an early age, or twin studies, would provide more definitive insights on potential neurodevelopmental underpinnings. Also, social stigma, as a potential cause of impaired social cognition or social awareness in some, should be measured in future studies. We used a modified version of the original SRS questionnaire in which four items had been removed, and several questions were reframed for adults. Since the items had been removed before administration, we could not compare the psychometrics of the instrument between the modified and original versions. Yet, similar trends were evident in the SRS subscales of social awareness and social communication where no items had been removed: for example, similar trends were found in the relationship between cortical thickness and SRS subscales or sex differences in these patterns. However, the findings that correlations between cortical thickness and social motivation subscale scores (in which two items had been removed) reached the statistically significant threshold only in one hemisphere, and/or that at-birth-assigned sex differences were the lowest for the social motivation subscale score, might have been affected by the modifications of this scale (Fig. 3 and Figure S2).

One frequently reported characteristic of individuals with ASD is hypersensitivity to sensory stimuli (e.g., odors, sounds, textures) (de Vries, 2021; Marco et al., 2011). The SRS does not capture these characteristics, and future studies are needed using different or multiple scales to capture this phenomenon to determine if it has a relationship with body incongruence. Moreover, future studies could investigate body incongruence, as measured, e.g., with the Body Morph Index, in individuals with ASD. It has already been shown that gender diversity and autistic traits are associated (Munoz Murakami et al., 2022), and investigating specific measures of gender dysphoria such as body incongruence in a sample of people with ASD would be complementary to this study. Finally, given the spectral nature shared by both ASD and gender dysphoria, employing dimensional measures proves beneficial in comprehending the correlation between these two conditions. While the SRS adeptly identifies the spectrums associated with ASD, there are limited analogous measures available for evaluating gender identity (Deogracias et al., 2007).

### Conclusion

Significantly higher autistic traits were evident in transgender participants compared with cisgender individuals. Higher autistic traits were associated with lower self-own-body congruence, however, not, when the correlation was tested in each separate group. The observed negative regional correlations between autistic traits and cortical thickness are patterns more consistent with ASD than gender dysphoria and could suggest that this cortical thinning could be secondary to the feeling of gender incongruence difficulties with social adjustments. Such a possibility should be tested further, for example, by employing longitudinal assessments before and after gender confirming treatment.

### Supplementary Information

Below is the link to the electronic supplementary material.

Supplementary file1 (DOCX 380 KB)

## Data Availability

Data from this study cannot be shared as the participants did not consent to data-sharing.
