# Supplementary material for: Gender Incongruence and Autistic Traits: Cerebral and Behavioral Underpinnings
Source: Arch Sex Behav. 2024 Feb 22;53(5):1873–84. doi: 10.1007/s10508-024-02809-5 (PMC11106115; doi:10.1007/s10508-024-02809-5)
Supplement: Supplementary file 1 — Supplementary file1 (DOCX 380 KB) [file 10508_2024_2809_MOESM1_ESM.docx]

Supplementary Materials

Box 1. Terminologies and their definitions

| Cisgender, Cis^1^ | A term used to refer to an individual whose gender identity aligns with the sex assigned to them at birth. The prefix cis- comes from the Latin word for “on the same side as.” People who are both cisgender and heterosexual are sometimes referred to as cishet (pronounced “sis-het”) individuals. |
| --- | --- |
| Transgender, Trans^1^ | Often shortened to trans, from the Latin prefix for “on a different side as.” A term describing a person’s gender identity that does not necessarily match their assigned sex at birth. Transgender people may or may not decide to alter their bodies hormonally and/or surgically to match their gender identity. This word is also used as an umbrella term to describe groups of people who transcend conventional expectations of gender identity or expression--such groups include, but are not limited to, people who identify as transsexual, genderqueer, gender variant, gender diverse, and androgynous. “Trans” is often considered more inclusive than transgender because it includes transgender, transsexual, transmasc, transfem, and those who simply use the word trans.  *Binary transgender* refers to those who identify as either man or woman. Non-binary transgender refers to those who identify as neither.  While not all transgender experience dysphoria, in this study we have focused on transgender individuals with gender dysphoria. For the sake of simplicity, throughout the document, transgender individuals refer to those with gender dysphoria. |
| Gender Dysphoria^2^ | The DSM-5-TR defines gender dysphoria in adolescents and adults as a marked incongruence between one’s experienced/expressed gender and their assigned gender, lasting at least 6 months, as manifested by **at least two** of the following:   - A marked incongruence between one’s experienced/expressed gender and primary and/or secondary sex characteristics (or in young adolescents, the anticipated secondary sex characteristics) - A strong desire to be rid of one’s primary and/or secondary sex characteristics because of a marked incongruence with one’s experienced/expressed gender (or in young adolescents, a desire to prevent the development of the anticipated secondary sex characteristics) - A strong desire for the primary and/or secondary sex characteristics of the other gender - A strong desire to be of the other gender (or some alternative gender different from one’s assigned gender) - A strong desire to be treated as the other gender (or some alternative gender different from one’s assigned gender) - A strong conviction that one has the typical feelings and reactions of the other gender (or some alternative gender different from one’s assigned gender)   In order to meet criteria for the diagnosis, the condition must also be associated with clinically significant distress or impairment in social, occupational, or other important areas of functioning. |
| Autistic traits^3^ | In addition to the individuals with ASD, many others exhibit subthreshold autistic or autistic-like traits, that is, problems or peculiarities in socio-communicative behavior, perception of others and self, and adaptation to the environment that do not meet formal criteria for an ASD. |
| Autism, or autism spectrum disorder (ASD)^2^ | refers to a broad range of conditions characterized by challenges with social skills, repetitive behaviors, speech and nonverbal communication.   1. Persistent deficits in social communication and social interaction across multiple contexts 2. Restricted, repetitive patterns of behavior, interests, or activities, as manifested by at least two of the following, currently or by history (examples are illustrative, not exhaustive) 3. Symptoms must be present in the early developmental period (but may not become fully manifest until social demands exceed limited capacities or may be masked by learned strategies in later life). 4. Symptoms cause clinically significant impairment in social, occupational, or other important areas of current functioning.   These disturbances are not better explained by intellectual disability (intellectual developmental disorder) or global developmental delay. |

^1^ Extracted from <https://vaden.stanford.edu/medical-services/lgbtqia-health/glossary-terms-related-transgender-communities>

^2^ The Diagnostic and Statistical Manual of Mental Disorders, Fifth Edition, Text Revision (DSM-5-TR)

^3^ Adapted from Lundstrom et al., (2012)


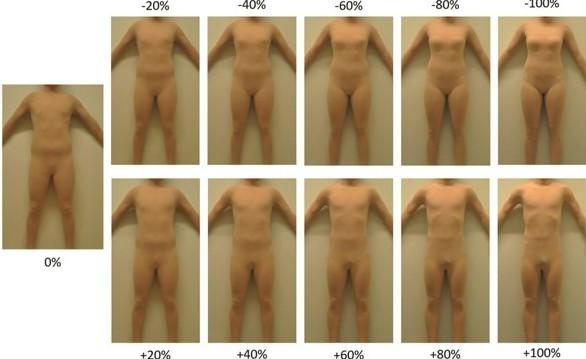


**Figure S1**. Examples of a male’s body images morphed, from left to right, to 20%, 40%, 60%, 80%, and 100% to the same (denoted by positive morph degrees) and the opposite (denoted by negative morph degrees) sex. Note that “100%” photographs were unaltered images of another person. Adapted from Burke et al., (2019).


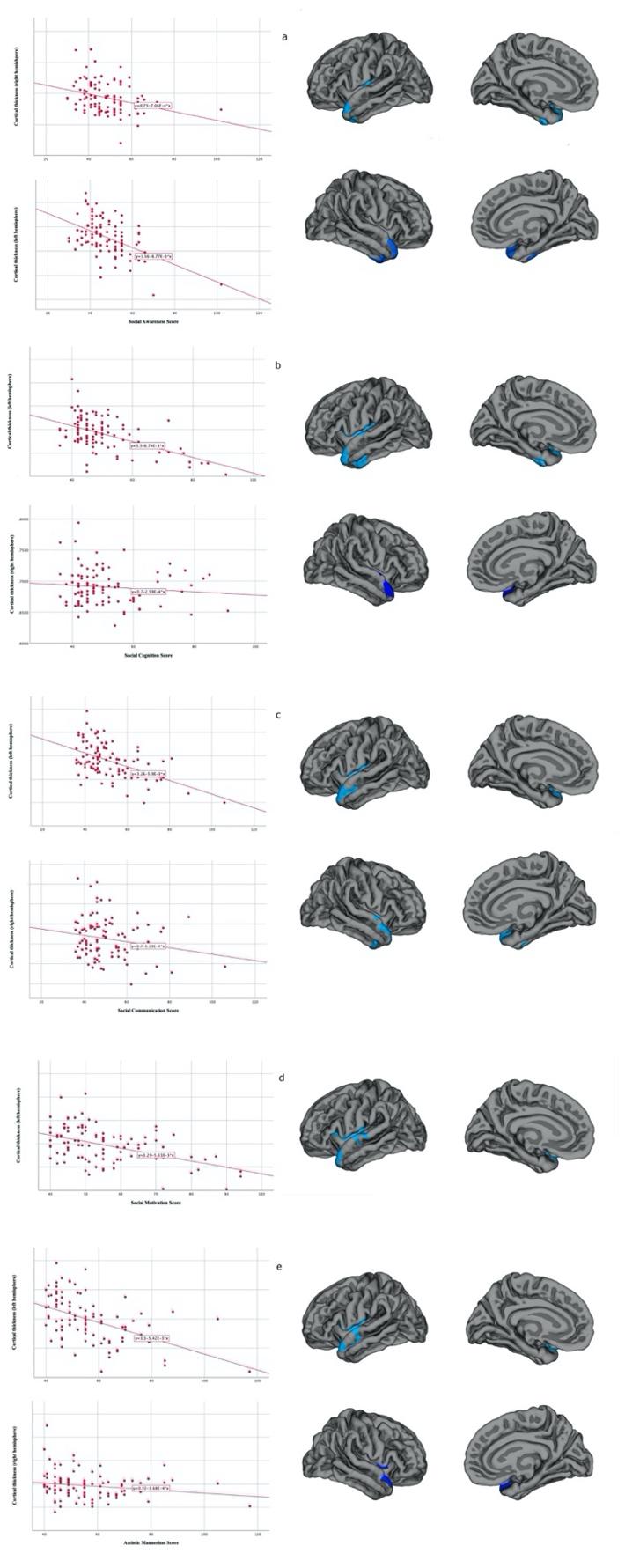


**Figure S2**. Correlation between Social Responsiveness Scale subscale scores and cortical thickness in various regions of brain; (a) social awareness; (b) social cognition; (c) social communication; (d) social motivation; (e) autistic mannerism. With slight variation, the significant clusters were in bilateral superior temporal gyri. The exception is for social motivation, which was significantly correlated with cortical thickness only in the left hemisphere. See Table S2 for the correlation coefficients and p values.


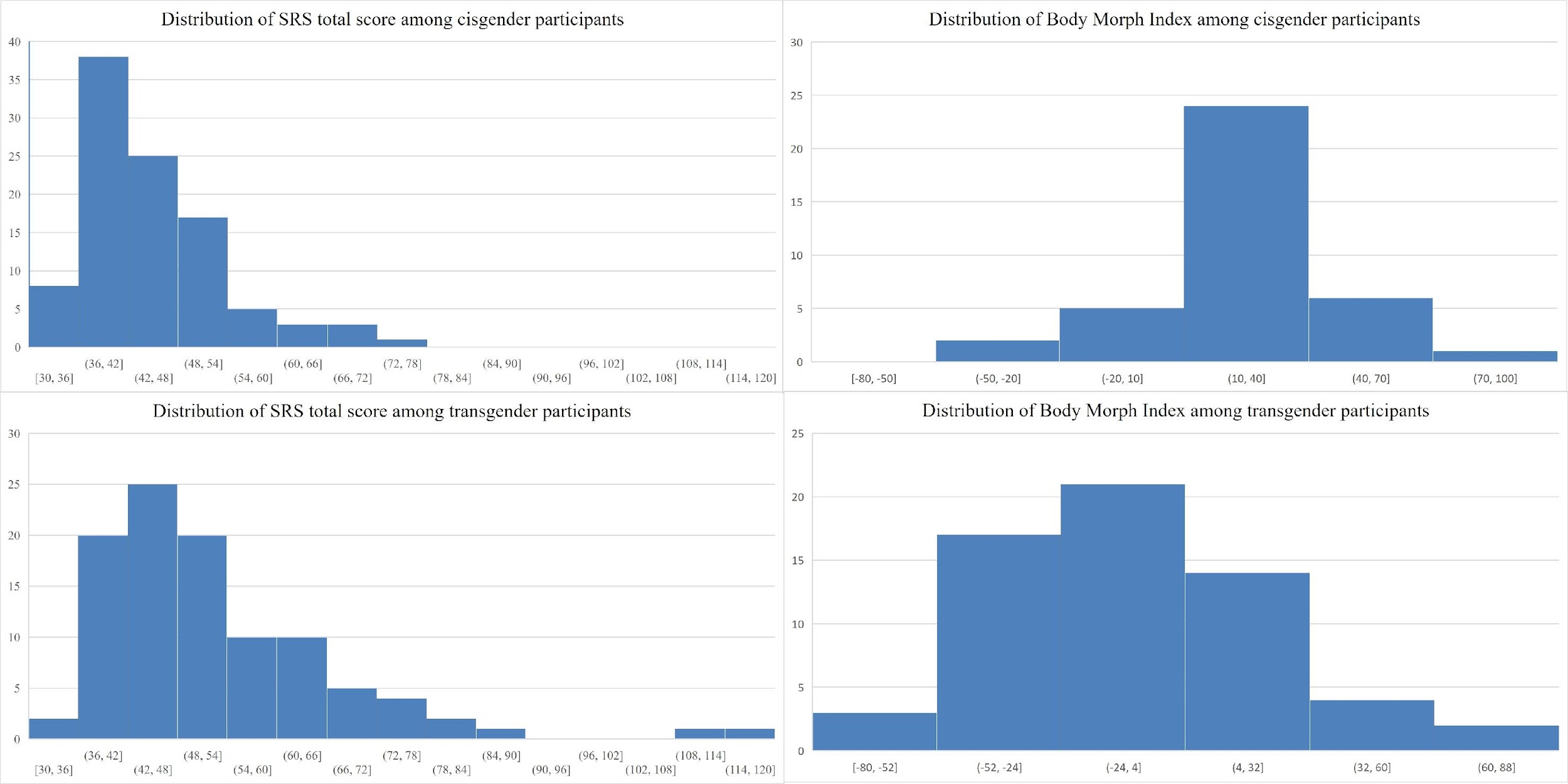


**Figure S3**. The distribution of Social Responsive Scale total scores and Body Morph Index among transgender and cisgender participants. Neither Social Responsiveness Scale total score nor any of its subscales were normally distributed, with a skewness of 1.23 (±.24) in cisgender participants and 1.68 (±.24) in transgender participants.

Table S1 Social Responsiveness Subscale scores

|  |  | Cisgender |  |  |  | Transgender | |  |  |
| --- | --- | --- | --- | --- | --- | --- | --- | --- | --- |
|  |  | Female |  | Male |  | Female |  | Male |  |
|  |  | KI | UCLA | KI | UCLA | KI | UCLA | KI | UCLA |
| Social Awareness | M (SD) | 45.34 (8.042) | 47 (8.496) | 41.51 (8.517) | 49.11 (9.867) | 48.02 (12.02) | 53.31 (9.818) | 45.94 (9.044) | 50.86 (7.819) |
|  | N | 41 | 12 | 37 | 9 | 48 | 13 | 31 | 7 |
| Social Cognition | M (SD) | 47.54 (8.364) | 48.75 (6.283) | 42.05 (4.618) | 50.11 (9.636) | 50.6 (10.88) | 52.08 (11.543) | 50.61 (12.758) | 49.14 (13.347) |
| Social Communication | M (SD) | 46.24 (8.549) | 46 (7.544) | 42.54 (5.586) | 51.22 (13.103) | 51.35 (13.695) | 52.62 (9.836) | 50.1 (12.557) | 48.14 (10.123) |
| Social Motivation | M (SD) | 46.9 (7.813) | 46.58 (6.908) | 45.54 (7.069) | 50.78 (6.723) | 54.6 (10.954) | 56.85 (11.901) | 56.81 (15.435) | 53.14 (16.728) |
| Autistic Mannerism | M (SD) | 50.85 (11.233) | 46.58 (5.551) | 46.27 (7.426) | 53.56 (16.801) | 56.25 (15.689) | 60.46 (12.087) | 53.1 (11.028) | 52.86 (14.781) |

Table S2 Social Responsiveness Scale total and subscale scores and cortical thickness correlations among transgender participants

| Correlations |  |  |  |  |  |  |  |
| --- | --- | --- | --- | --- | --- | --- | --- |
| Spearman's rho |  | Total SRS | Social Cognition | Social Awareness | Social Communication | Social Motivation | Autism Mannerism |
| Cortical Thickness (Right)^1^ | Correlation Coefficient | -.18 | -.07 | -.32** | -.14 | -.41** | -.18 |
|  | Sig. (2-tailed) | .069 | .5 | .001 | .15 | <.001 | .07 |
|  | N | 99 | 99 | 99 | 99 | 99 | 99 |
| Cortical Thickness (Left)^2^ | Correlation Coefficient | -.50** | -.43** | -.45** | -.40** |  | -.51** |
|  | Sig. (2-tailed) | 0 | 0 | 0 | 0 |  | 0 |
|  | N | 99 | 99 | 99 | 99 |  | 99 |
| ** Correlation is significant at the 0.01 level (2-tailed).  * Correlation is significant at the 0.05 level (2-tailed). | | | | | | | |

^1&2^ These are mean cortical thickness in regions in which an association between cortical thickness and Social Responsive Scale scores have been found.

Table S3 Correlation coefficient between Social Responsiveness Scales and its subscales with Body Morph Index (short and long exposure) among all participants

| Social Responsiveness Scale Total Score | Pearson Correlation | -.325** | -.241* |
| --- | --- | --- | --- |
|  | N | 95 | 94 |
| Social Cognition | Pearson Correlation | -.260* | 1 |
|  | N | 95 | 94 |
| Social Communication | Pearson Correlation | -.262* | -0.202 |
|  | N | 95 | 94 |
| Social Motivation | Pearson Correlation | -.389** | -.205* |
|  | N | 95 | 94 |
| Autistic Mannerism | Correlation Coefficient | -.274** | -0.179 |
|  | N | 94 | 94 |
| Social Awareness | Correlation Coefficient | -0.108 | -0.094 |
|  | N | 94 | 94 |

** <.01; * < .05
